# Visual Experience Determines the Use of External Reference Frames in Joint Action Control

**DOI:** 10.1371/journal.pone.0059008

**Published:** 2013-03-11

**Authors:** Thomas Dolk, Roman Liepelt, Wolfgang Prinz, Katja Fiehler

**Affiliations:** 1 Department of Psychology, Max-Planck-Institute for Human Cognitive and Brain Sciences, Leipzig, Germany; 2 Institute for Psychology, University of Muenster, Muenster, Germany; 3 Department of Psychology, Justus-Liebig-University Giessen, Giessen, Germany; Goldsmiths, University of London, United Kingdom

## Abstract

Vision plays a crucial role in human interaction by facilitating the coordination of one's own actions with those of others in space and time. While previous findings have demonstrated that vision determines the default use of reference frames, little is known about the role of visual experience in coding action-space during joint action. Here, we tested if and how visual experience influences the use of reference frames in joint action control. Dyads of congenitally-blind, blindfolded-sighted, and seeing individuals took part in an auditory version of the social Simon task, which required each participant to respond to one of two sounds presented to the left or right of both participants. To disentangle the contribution of external—agent-based and response-based—reference frames during joint action, participants performed the task with their respective response (right) hands uncrossed or crossed over one another. Although the location of the auditory stimulus was completely task-irrelevant, participants responded overall faster when the stimulus location spatially corresponded to the required response side than when they were spatially non-corresponding: a phenomenon known as the social Simon effect (SSE). In sighted participants, the SSE occurred irrespective of whether hands were crossed or uncrossed, suggesting the use of external, response-based reference frames. Congenitally-blind participants also showed an SSE, but only with uncrossed hands. We argue that congenitally-blind people use both agent-based and response-based reference frames resulting in conflicting spatial information when hands are crossed and, thus, canceling out the SSE. These results imply that joint action control functions on the basis of external reference frames independent of the presence or (transient/permanent) absence of vision. However, the type of external reference frames used for organizing motor control in joint action seems to be determined by visual experience.

## Introduction

Joint action plays a fundamental role in human life. It requires the coordination of one's own actions with those of others in space and time and across different sensory modalities [1]–[2]. Findings from animal [3]–[4], human [5]–[6], and modeling studies [7]–[8] suggest that visual, auditory, and tactile targets are represented in a common visual reference frame that facilitates communication and integration of different sensory inputs and enables the translation into movement plans. As a consequence, vision seems to be critically involved in coding action-space. This is supported by studies with blind and sighted people demonstrating that developmental vision determines the default use of reference frames. While late-blind and sighted people preferably code space in external, environmental-centered coordinates, congenitally-blind people favor the use of internal, observer-centered coordinates [9]–[10]. Yet, it is still unclear if and how visual experience influences the use of reference frames for organizing motor control in joint action. This appears particularly relevant since interacting with others in space and time changes the contextual demand of action control, which may lead to a stronger contribution of external, environmental-centered reference frames. To coordinate one's own actions with those of others, each of the agents has to plan, execute, and monitor their own actions and, in addition, has to observe, understand, and anticipate the other persons' actions in the context of a rapidly changing environment. This requires a continuous update of spatial information whenever the point of reference moves in space. Because our eyes, head, and/or body frequently change position, the use of such internal, observer-centered reference frames are computationally more costly than the use of a more stable external, environmental-centered reference frame; particularly when the spatial information of more than one person needs to be considered. For individual action control, internal and external reference frames might be equally successful; however, for joint action control external, environmental-centered reference frames are likely to be more effective.

One of the most prominent paradigms for studying joint action control is the social Simon task in which two people share the standard version of the Simon task. In the standard Simon task, participants carry out spatially defined responses (e.g., using left or right key presses) to non-spatial stimulus attributes (e.g., auditory pitch or visual color) that randomly appear to the left or right of the participant. For example, participants are instructed to press a right key whenever they perceive a high-pitched tone and a left key in response to a low-pitched tone. Although stimulus location is completely task-irrelevant, responses are faster when the stimulus location spatially corresponds to the required response side; a phenomenon known as the (standard) *Simon effect* ([11]; see [12] for a review). According to the dimensional overlap model, the Simon effect is explained by a match between the spatially irrelevant dimension of the stimulus and the relevant response dimension [13]–[14]. It is assumed that responses are automatically activated if the location of the stimulus matches the location of the correct response, facilitating task performance, whereas a spatial mismatch between stimulus–response (S–R) pairs leads to response competition, resulting in longer reaction times. However, the Simon effect is usually not observable when a single person exclusively operates the left key in response to only one of the two stimulus attributes, rendering the task a go-nogo task [15]. If the same go-nogo task is shared between two people, so that each of them responds to their assigned stimulus by operating one of the two response keys (e.g., the participant on the left presses the left response key whenever his/her stimulus appears, whereas the participant on the right responds by pressing the right key), a joint S-R compatibility effect is elicited [16], known as the *social Simon effect* (*SSE*). Similar to the standard Simon effect observed when one participant is responsible for both responses, participants in the social Simon task respond faster when the location of the assigned stimulus spatially corresponds to the correct response side (i.e., to the responsible agent).

The SSE has been explained by the co-representation of the co-actor's action, which is assumed to be a social, automatic, and mandatory process (*action co-representation account*; [16]–[18]). Accordingly, it has been demonstrated that an SSE emerges when a person interacts (or believes they are interacting) with another intentional agent, but not when they interact with a puppet or computer [16], [19]–[21]. Such effective modulations appear to be relatively independent of sensory feedback. When participants were pre-instructed about their own and others' responsibilities in a social Simon task, neither visual nor auditory feedback was necessary for an SSE to emerge [16], [22].

However, recent findings have challenged the action co-representation account [23]–[26]. For example, individuals with autism, who are assumed to have deficits in processing social information [27]–[28], showed a normal SSE [29], which speaks against the idea that high-level representational processing (e.g., inferring mental states of others, such as their beliefs, thoughts, desires, or intentions; [30], [31]) necessarily underlies the SSE [23]–[24], [32]–[33]. Similarly, Guagnano and colleagues [26] recently claimed that the co-actors' actions may provide a spatial reference frame. Accordingly, an actor codes his/her own action events in relation to the action events of a co-actor—just like when one's own left or right actions in the standard Simon task provide a spatial reference for relative response coding [15]. Most recent findings on the SSE, however, extended these assumptions by showing that neither the active participation in the task nor the physical presence of a co-actor is necessary to elicit an SSE [23]. Hence, as long as social or non-social action events attract attention, these events seem to be represented irrespective of whether another agent or an inanimate object produces these events (*referential coding account*; [24]). The referential coding account provides a theoretical, ideomotorically inspired framework that is capable of integrating a broad range of results of social and non-social go-nogo Simon effects: The presence of alternative (social or non-social) action events (or perceivable effects thereof) requires making a discrimination between the cognitive representation referring to one's own action and all concurrently activated event representations, which can be achieved by referential coding—the spatial coding of one's action relative to other external events. This intentional weighting of response alternatives [14], [34] leads, in turn, to matches or mismatches of spatial S-R features—a necessary condition for Simon effects to emerge.

It is still a matter of debate as to whether participants in the social Simon task encode their responses in reference to the location of the other responding agent (agent-based coding) or in reference to the location of the response keys (response-based coding). In contrast to the standard Simon task in which a single participant may respond with their left and right hand, in the social Simon task a dyad of participants sits next to each other and each participant responds exclusively with one hand; usually the right hand. Hence, in the social Simon task, the spatial origin of the agents' bodies and the spatial origin of the response keys provide two external, environmentally-based frames of reference: an agent-based frame and a response-based frame. If participants perform the social Simon task with each of their respective response (right) hands uncrossed over one another (i.e., in the same left-right organization as their bodies, with respect to one another), both external reference frames (agent-based and response-based coordinates) are aligned (spatially compatible). In contrast, when participants cross their respective response (right) hands over one another, i.e., the left sitting person operates the right response key and vice versa, agent-based and response-based coordinates are misaligned (spatially incompatible). Addressing this issue, Welsh found an SSE for uncrossed and crossed hand positions irrespective of whether the participants performed the visual version of the social Simon task with their inner (right/left) or outer (left/right) hands [35]. This result suggests that the SSE is neither dependent on the spatial origin of the responding agents (i.e., external, agent-based coordinates) nor the anatomical origin of the responding hands (i.e., internal, observer-centered coordinates), but rather it is tied to the spatial location of the response keys (i.e., external, response-based coordinates). However, recent findings from trial-by-trial analyses argue for a more flexible coding strategy that switches between both external (the agent-based and the response-based) reference frames, depending on the compatibility of the previous trial, i.e., whether trials had changed from compatible to incompatible or visa versa [36]. Here, opposing SSEs of roughly the same size, i.e., a positive SSE following compatible trials (response-based coding) and a negative SSE after incompatible trials (agent-based coding), led to the cancellation of an overall SSE.

Because spatial coding of action-specific information is tightly linked to vision, comparison of congenitally-blind and sighted people may provide a fruitful approach to shed more light on the underlying reference frame/s used for joint action control. Using an auditory version of the standard Simon task, Röder and colleagues [9] showed that sighted and late-blind participants responded faster when the location of the stimulus and the response key was congruent than incongruent (i.e., Simon effect), irrespective of whether hands were crossed or uncrossed. Congenitally-blind participants, in contrast, showed the reversed (negative) Simon effect when hands were crossed, i.e., they responded faster when the location of the stimulus (e.g., right) and the anatomical origin of the responding hand (e.g., right) were congruent and, thus, the location of stimulus and response-key were incongruent [9]. Hence, congenitally-blind people seem to code the stimulus location with respect to their left or right hand (internal, observer-centered coordinates), regardless of the location of the response keys, whereas people with visual experience (sighted and late-blind) preferentially code the location of the stimulus with respect to the location of the response keys (using external, response-based coordinates; see also [37]-[39], but see [40] for evidence of internal coding). Similar results have been reported recently in a goal-directed reaching task [10]. Note, that these results do not imply that congenitally-blind individuals are incapable of successfully using external reference frames, but rather point to a default use of an internal, observer-centered reference frame in individual manual tasks (e.g., [41]–[42]).

While it appears to be a reasonable strategy to rely either on external, environmental, or internal, observer-centered coordinates in individual manual tasks, joint task performance changes the contextual demand of action control and enhances the relevance of external, environmental-centered reference frames. Such a common external reference frame could facilitate the communication and coordination between people in joint action tasks by providing a common representational ground. Since sighted and congenitally-blind people are both able to efficiently interact with others, it is conceivable that they rely on external reference frame/s (i.e., agent-based and/or response-based) when they engage in social manual tasks. However, if vision is crucially involved in the use of external spatial coordinates in joint action, as has been shown for individual action tasks [9]–[10], the use of external reference frame/s in social manual tasks may differ between sighted and congenitally-blind people. There is evidence that congenitally-blind people compensate for the lack of visual input with their remaining senses, to some extent (*compensatory hypothesis*, [43]). A more extensive use of these senses can result in superior perceptual and spatial skills, as well as more efficient attentional processes [43]–[44]. In contrast to the response buttons, a responding co-actor in the social Simon task can provide direct auditory and tactile feedback about his/her location in space, which offers a reliable spatial reference (e.g., by crossing participants' arms over one another) and, thus, may facilitate agent-based coding. We assume that congenitally-blind individuals rely more strongly on an agent-based reference frame in the social Simon task compared to sighted individuals because the co-actor provides a perceptually rich and salient source of non-visual spatial information that is diminished in response-based coding (e.g., no tactile feedback about the location of the co-actor's response key).

Here, we studied dyads of congenitally-blind participants with no experience of visual input, blindfolded-sighted participants, and seeing participants in an auditory version of the social Simon task. In order to investigate if and how visual experience influences the use of external reference frame/s for organizing motor control in joint action, participants performed a social Simon task using their right hand under uncrossed and crossed hand conditions. To that end, participants operated the response key in front of their own body in the uncrossed hand condition and the response key in front of the co-actor's body in the crossed hand condition. If joint action initiates or even requires the use of external coordinates, uncrossed hands should facilitate joint task performance, given that both the agent-based and the response-based reference frames are spatially aligned. In contrast, when hands are crossed a mismatch of the agent-based and response-based reference frames may cause a conflict, thereby altering joint task performance. Based on previous findings regarding the SSE in seeing individuals [16], [22], [35], we expected an overall positive SSE independent of hand position and visual feedback during task performance in both sighted groups (blindfolded and seeing), implying the use of a response-based reference frame. Following our hypothesis that congenitally-blind participants use external reference frame/s when interacting with others, we predicted there would be a positive SSE when hands were uncrossed. The hand-crossed condition where the agent-based and response-based reference frames are misaligned (spatially incompatible) was intended to test our hypothesis that lack of vision influences the use of external reference frames. If visual experience has an effect on external coding strategies for organizing motor control in joint action, we would expect differences in the SSE between congenitally-blind and sighted participants in the hand-crossed condition. While a negative SSE would suggest that congenitally-blind people mainly rely on an agent-based reference frame, a reduced, or even missing SSE, would imply a flexible switch between agent-based and response-based coding strategies in order to reduce conflicting response tendencies when both external reference frames are spatially misaligned.

## Materials and Methods

### Participants

A group of congenitally-blind individuals with no history of neurological or hearing problems and two groups of sighted adults without any history of medical issues (neurological or sensorial) participated in the present study. All participants were naive with regard to the hypothesis of the experiment, gave their written consent, and were paid for their participation. The study was conducted in accordance with the Declaration of Helsinki and was approved by the local ethics committee of the University of Marburg.

The congenitally-blind group consisted of 16 adults (10 female; 16–34 years of age; *M* = 22.8, *SD* = 5.7). All congenitally-blind participants, except one, were right-handed, as assessed by the Edinburgh Handedness Inventory (EHI; [45]). 14 participants were totally blind (*n* = 14), whereas the remaining two had rudimentary sensitivity for brightness differences without any pattern vision. Blindness was, in all cases, due to peripheral reasons (see Table 1 for details).

**Table 1:** Description of the congenitally-blind participants.

Pts	Age (years)	Sex	EHI score	MWT-B score	Age of blindness	Cause of blindness	Education
1	29	M	64	130	birth	DDT during pregnancy	US
2	34	M	100	130	birth	retinitis pigmentosa	UD
3	27	M	82	118	prenatal	VI during pregnancy	US
4	23	M	54	118	birth	retinitis pigmentosa	US
5	31	F	100	130	birth	retrolentale fibroplasie	US
6	31	F	27	136	birth	retrolentale fibroplasie	UD
7	16	F	40	100	birth	nervus opticus defect	HS
8	19	F	100	104	birth	retinotpathy of prematurity	HS
9	20	F	30	97	birth	LCA	HS
10	18	F	82	118	birth	gene defect	HS
11	19	F	91	118	birth	retinotpathy of prematurity	HS
12	18	M	50	118	birth	retinotpathy of prematurity	HS
13	20	M	56	104	birth	nervus opticus defect	HS
14	18	F	50	112	birth	retinotpathy of prematurity	HS
15	21	F	−100	112	birth	retinotpathy of prematurity	HD
16	20	F	54	101	birth	gene defect	HD

*Note.* The handedness score was assessed by the Edinburgh Handedness Inventory (EHI; right-handed: maximum score +100; left-handed: maximum score−100; [45]). The MWT-B [46] is a general measure of the participants' cognitive capacity (IQ). DDT, Dichloro-Diphenyl-Trichloroethane; F, female; HD, High school degree; HS, High school student; LCA, Leber Congenital Amaurosis; M, male; Pts, Participants; UD, University degree; US, University Student; VI, Virus Infection.

One half of the seeing controls (*n* = 16; 10 female; 20–34 years of age; *M* = 23.9, *SD* = 4.1) served as the blindfolded-sighted group and the other half (*n* = 16; 10 female; 20–33 years of age; *M* = 23.3, *SD* = 4.0) served as the sighted control group. The participants in both groups were matched to the congenitally-blind in age(±6 years), gender, IQ (as assessed by the MWT-B; [46]) and handedness.

### Task and statistical analysis

**Figure 1:**
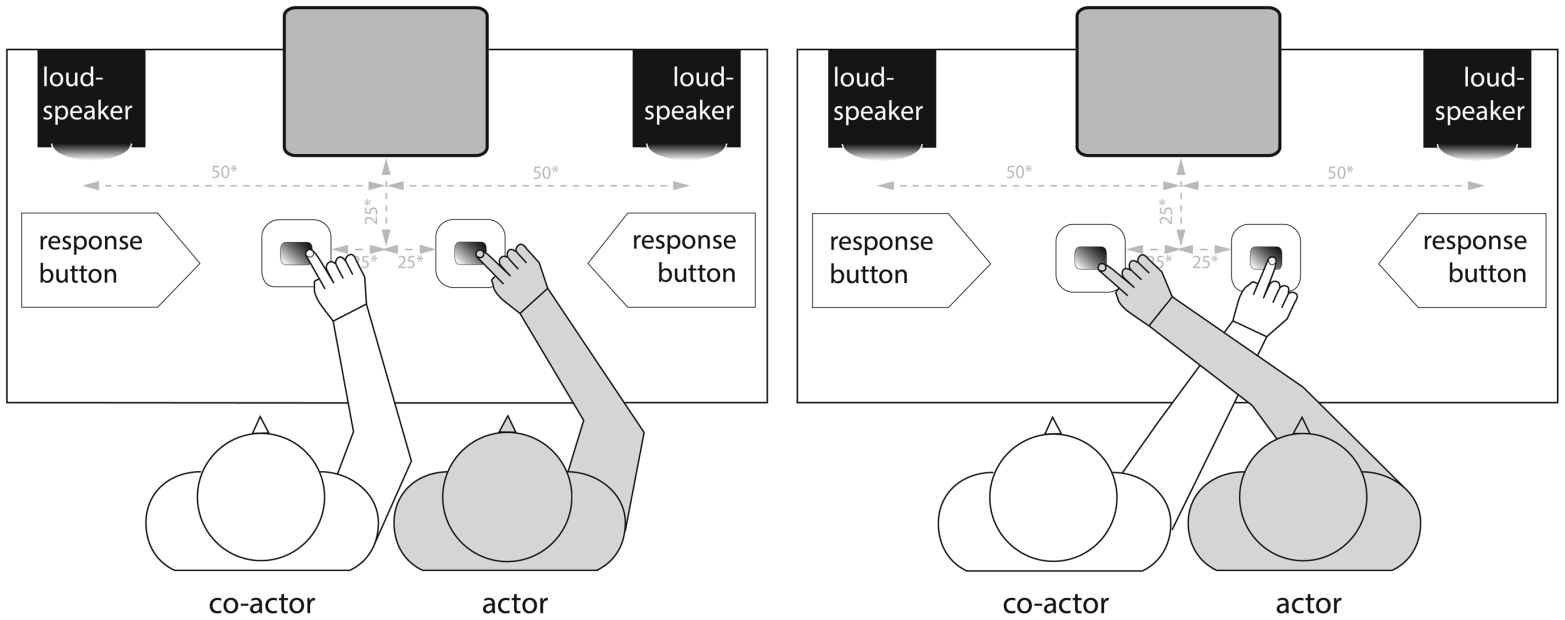
Experimental Design. Gray shaded participant depicts the actor, while the white shaded participant depicts the co-actor. Participants performed the Joint Simon task under ‘hands uncrossed’ (left panel) and ‘hands crossed’ (right panel) conditions. Gray dashed arrows illustrate the distance between loudspeaker and response buttons in cm (*).

Two acoustic signals (sound A and sound B) were used as go and no-go stimuli in an auditory social Simon task. The two sounds consisted of spoken Dutch color words: ‘groen’ (*green*) and ‘paars’ (*purple*) that were played in reverse at approximately 60dB, leading to two easily distinguishable acoustic signals: ‘oerg’ (mean pitch = 135.5 Hz) and ‘chap’ (mean pitch = 105.2 Hz). In each trial, one of the two sounds was presented via either the left or the right loudspeaker. The loudspeakers were separated by a distance of one meter (see Fig 1). Thus, as can be seen in Figure 1, the location from which each acoustic signal appeared was either to the left or the right of both participants.

Prior to the initial instruction/training phase of the experiment, participants were seated next to each other (position left or right was counterbalanced across participants in each group), either side of the midline of a computer monitor. Hence, both participants in each dyad had an equal visual status (either congenitally-blind, blindfolded-sighted or seeing). In the ‘hands uncrossed’ condition participants were asked to place their right index finger on a response button 25 cm in front and 25 cm to the left or right of the midline of a computer monitor, while placing their left hand underneath the table on their left thigh (Fig. 1, left panel). In contrast, in the ‘hands crossed’ condition the participant on the right was asked to place their right index finger on the left response button, whereas the participant on the left was asked to put their right index finger underneath the arm of the right participant and on the right response button (Fig. 1, right panel).

To familiarize participants with the task, there was an instruction/training phase(∼5 min) prior to the experiment that included familiarization with the two sounds (A and B) and their assignment as go and nogo's, and a training of 8 sample trials. After the instruction/training phase was completed, the experimental phase started with either the ‘hands uncrossed’ or the ‘hands crossed’ condition; the order was counterbalanced across subjects.

There were two blocks (1 hands crossed, 1 hands uncrossed) of 64 trials for each go and nogo-signal (32 with a spatially compatible S-R relationship and 32 with a spatially incompatible S-R relationship, randomly presented within each block). Each trial began with the presentation of a warning sound for 300 ms. After 1000 ms, the critical sound—either signal A or B—was presented for 300 ms to the right or the left side of both participants, who were instructed to respond as quickly and as accurately as possible to their individual target signal (either signal A or B, balanced across subjects). After a response was given or 1700 ms had passed, a 1000 ms inter-stimulus interval (ISI) followed. The whole experiment took approximately 40 min.

In accordance with previous studies [9], [32], we excluded all trials in which responses were incorrect (1.1%), faster than 150 ms, or slower than 1000 ms (0.2%) from statistical analysis. In both conditions (hands crossed, hands uncrossed) responses were coded as compatible and incompatible with respect to the spatial relation of stimulus and response key position. In order to test the hypothesis that congenitally-blind participants differ from seeing and blindfolded-sighted participants in the crossed or uncrossed hand conditions, we calculated the following analyses: Correct RTs were submitted to a 2×2×2 mixed analysis of variance (ANOVA) with the within-subjects factors Compatibility (compatible, incompatible) and Condition (hands crossed, hands uncrossed) and the between-subjects factor Visual Status (congenitally-blind, blindfolded-sighted, seeing). The same ANOVA was conducted for the congenitally-blind and blindfolded-sighted groups to control for the effect of online vision during task performance. To test whether compatibility (compatible, incompatible) varies with hand position (hands crossed, hands uncrossed) within each of the three groups (congenitally-blind, blindfolded-sighted, and seeing), we unpacked the three-way interaction by applying 2×2 within-subjects ANOVAs. Post-hoc *t*-tests were then calculated and corrected for multiple comparisons using Bonferroni correction. For completeness, we also report the results of the overall 2×2×3 mixed ANOVA with the factors Compatibility (compatible, incompatible), Condition (hands crossed, hands uncrossed) and Visual Status (congenitally-blind, blindfolded-sighted, seeing).

## Results

### Reaction times

Responses were overall faster when stimulus location and response key position were spatially compatible (mean RT = 338 ms *SE*±9.2) than incompatible (mean RT = 352 ms *SE*±9.8), i.e., an SSE occurred (Compatibility, *F*_1,45_ = 55.76, *P*<0.001, *η*_p_^2^ = 0.55). More importantly, depending on the visual status (congenitally-blind, blindfolded-sighted, seeing), the SSE varied with hand position (hands crossed, hands suncrossed), as indicated by a significant three-way interaction of Compatibility×Condition×Visual Status, *F*_2,45_ = 4.32, *P* = 0.02, *η*_p_^2^ = 0.16.

**Figure 2:**
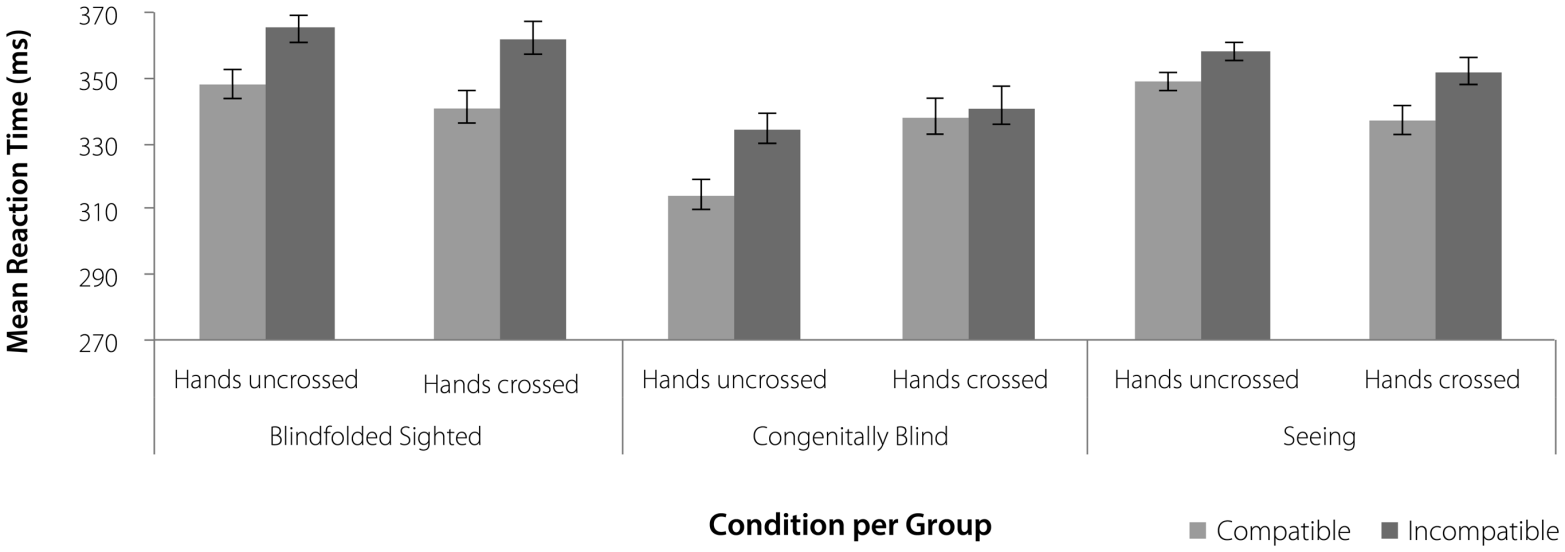
Results. Mean reaction time as a function of group (Blindfolded-Sighted, Congenitally-Blind, Seeing), condition (hands uncrossed, hands crossed) and spatial stimulus–response compatibility (Compatible, Incompatible). Error bars depict the standard error of the mean difference.

With the first planned comparison, we examined the SSE of congenitally-blind and seeing participants under crossed and uncrossed hand conditions. As expected, we found a significant 3-way interaction (Compatibility×Condition×Visual Status, *F*_1,30_ = 6.10, *P* = 0.02, *η*_p_^2^ = 0.17) indicating that the SSE differs for different hand positions, depending on the group (see Fig. 2). The second planned comparison was carried out within the congenitally-blind and the seeing groups and tested for differences of the SSE as a function of hand position. The results demonstrated that the SSE varied for crossed and uncrossed hands in the congenitally-blind group (Compatibility×Condition, *F*_1,15_ = 4.82, *P* = 0.04, *η*_p_^2^ = 0.24), but not in the seeing group (Compatibility×Condition, *F*_1,15_ = 1.38, *P* = 0.26, *η*_p_^2^ = 0.08), who showed an SSE irrespective of hand position (Compatibility, 12 ms, *F*_1,15_ = 23.08, *P*<0.001, *η*_p_^2^ = 0.61). Post-hoc *t*-tests revealed that congenitally-blind participants showed a significant SSE when hands were uncrossed (20 ms, *t*(15) = 4.19, *p_Bonferroni corrected_* = 0.001), but not when hands were crossed (3 ms, *t*(15) = 0.57, *p_Bonferroni corrected_* = 0.58).

In order to control for online visual feedback during task performance, we carried out the same analyses for congenitally-blind and blindfolded-sighted participants. Here, we obtained similar findings as for the comparison of congenitally-blind and seeing participants (see Fig. 2), i.e., there was a significant 3-way interaction (Compatibility×Condition×Visual Status, *F*_1,30_ = 5.49, *P* = 0.03, *η*_p_^2^ = 0.16) that resulted from variation of the SSE with hand position in congenitally-blind people (Compatibility×Condition, *F*_1,15_ = 4.82, *P* = 0.04, *η*_p_^2^ = 0.24), which was absent in blindfolded-sighted people (Compatibility×Condition, *F*_1,15_ = 0.93, *P* = 0.35, *η*_p_^2^ = 0.06). Blindfolded-sighted participants demonstrated an SSE independent of hand position (19 ms, *F*_1,15_ = 27.04, *P*<0.001, *η*_p_^2^ = 0.64).

**Figure 3:**
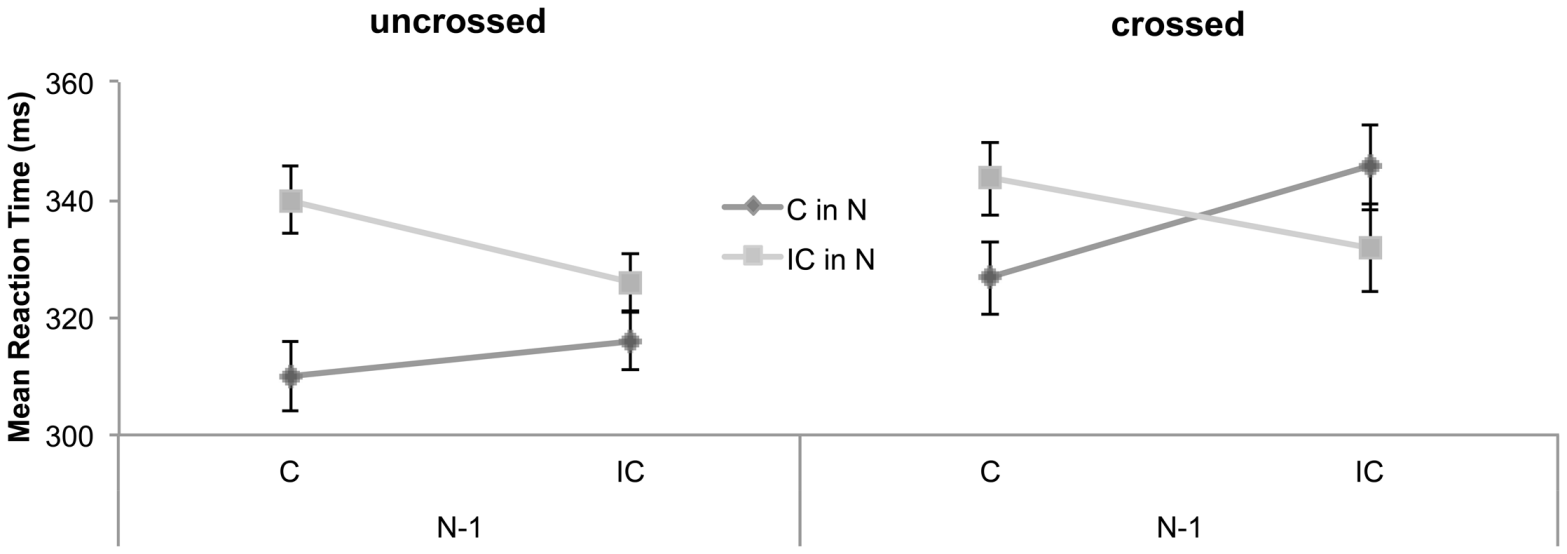
Sequential trial-by-trial dependencies. Mean reaction time in milliseconds (ms) of the congenitally-blind participants as a function of compatibility in trial N, depending on the compatibility (C: Compatible and IC: Incompatible) of the previous trials (N–1) for the uncrossed hand (left panel) and the crossed hand condition (right panel). Error bars depict the standard error of the mean difference.

To gain a better understanding of the dynamics mediating the SSE in the congenitally-blind participants, we analyzed the sequential trial-by-trial dependencies in both conditions (i.e., crossed and uncrossed hands) separately. A 2×2 ANOVA with the within-subjects factors Compatibility in preceding Trial _N–1_ (compatible, incompatible) and Compatibility in current Trial _N_ (compatible, incompatible) revealed a significant interaction between Compatibility _N–1_×Compatibility _N_ for uncrossed hands, *F*_1,15_ = 10.12, *P* = 0.006, *η*_p_^2^ = 0.40, and for crossed hands, *F*_1,15_ = 14.21, *P* = 0.002, *η*_p_^2^ = 0.49, indicating a sequential modulation of the SSE in both conditions. For the hands uncrossed condition, further analyses showed an SSE after compatible trials (30 ms, *t*(15) = 5.05, *p_Bonferroni corrected_*<0.001) and a trend for an SSE after incompatible trials (10 ms, *t*(15) = 1.94, *p_Bonferroni corrected_* = 0.07; see Fig. 3). For the hands-crossed condition, an SSE was found after compatible trials (17 ms, *t*(15) = 2.53, *p_Bonferroni corrected_* = 0.02) and a trend for a negative SSE (faster responses for incompatible than compatible trials) after incompatible trials (-14 ms, *t*(15) = 1.95, *p_Bonferroni corrected_* = 0.07; see Fig. 3).

### Error rates

We observed higher error rates for incompatible trials (1.5%) than for compatible trials (0.8%), which were comparable for crossed and uncrossed hand positions and consistent across all three groups of subjects (main effect of Compatibility, *F*_1,45_ = 4.71, *P* = 0.04, *η*_p_^2^ = 0.10). No further main effects or interactions were found (all *Ps*>0.05).

## Discussion

The present study investigated the role of visual experience in setting up spatial reference frames in joint action control. Using an auditory version of the social Simon task with hands crossed and uncrossed, we found that the social Simon effect (SSE) was unaffected by hand position in dyads of sighted (blindfolded and seeing) participants and independent of online visual feedback during the task; suggesting a response-based coding strategy. In contrast, dyads of congenitally-blind participants showed an SSE when hands were uncrossed, but the SSE was absent with crossed hands. While sighted people primarily rely on a response-based reference frame, congenitally-blind people seem to combine agent-based and response-based coordinates. These results suggest that joint action control functions on the basis of external coding strategies in both sighted (blindfolded and seeing) and congenitally-blind individuals. However, the type of external reference frame/s used for organizing motor control in joint action seems to be determined by visual experience.

Here, we demonstrate for the first time that an auditory SSE occurs in blind individuals who have never experienced visual input in their life. Furthermore, we also observed an SSE in blindfolded-sighted and seeing individuals who only differed in the amount of visual feedback during task performance. Indeed, it has been shown that vision substantially contributes to the coordination of shared actions and the understanding of others' intentions [47]–[49]. For example, there is evidence that the observation of others' gaze direction facilitates nonverbal communication in cooperative interaction [50] and changes the control of motor acts on the behavioral level [51]–[52], as well as on the cortical level [53]–[55]. Our findings extend these results and suggest that joint action control, as measured by the SSE, also functions without online visual feedback and develops independently of visual input during life.

The present finding of an overall positive SSE (as a result of S–R compatibility coding with respect to the location of the response keys) in blindfolded-sighted and seeing subjects for crossed and uncrossed hands is in line with previous findings on the auditory SSE [22] and suggests that joint action control in sighted individuals relies primarily on an external response-based reference frame. However, results from visual social Simon tasks with crossed and uncrossed hands seem to be less consistent. While the study of Welsh [35] provided further evidence for a response-based coding strategy, more recent results from Liepelt and colleagues [36] suggested a more flexible use of agent-based and response-based coding strategies depending on the compatibility of the previous trial.

In contrast to the findings in sighted participants, we found that joint task performance of congenitally-blind participants varied with hand position. They showed an SSE with uncrossed hands, which was absent when the hands of the two participants were crossed. That is, response times for compatible and incompatible trials were comparably slow when congenitally-blind participants sitting on the right operated the left key and vice versa. Thus, the information processing advantage obtained with uncrossed hands (i.e., being faster in compatible than incompatible trials when the location of the response key and the personal position were spatially aligned) was significantly reduced when a conflict between the agent-based and response-based coordinate systems was introduced by crossing of the hands. Therefore, in congenitally-blind individuals action facilitation due to S-R congruency was only evident when both external reference frames were spatially aligned, i.e., when agent-based and response-based coding worked in the same direction. Accordingly, neither the use of a pure agent-based reference frame (reflected by an overall negative SSE) nor a pure response-based reference frame (reflected by an overall positive SSE) can reasonably explain the results pattern of the congenitally-blind participants.

An additional analysis of the sequential trial-by-trial dependencies for the congenitally-blind participants for crossed hands revealed a positive SSE when the preceding trial was compatible (response-based coding) and a negative SSE when the preceding trial was incompatible (agent-based coding). Since both effects were roughly of the same size, the positive and the negative SSEs canceled each other out. Consistent with the conflict adaptation theory [56], the present findings suggest that the congenitally-blind individuals may have used a combination of agent-based and response-based external reference frames and flexibly switched between the two coding schemes to reduce conflict on subsequent trials (for a more detailed discussion and an alternative binding explanation see [36]). Taken together, the findings of the trial-by-trial analysis indicate that task performance of the congenitally-blind individuals was facilitated if the spatial compatibility of either agent-based or response-based coding was preserved from the preceding to the current trial, whereas performance was impaired, when the subsequent trial involved a change of the spatial reference frame.

As outlined above, the present results imply that congenitally-blind people, in contrast to sighted people, may use an agent-based reference frame in addition to response-based coding. Due to their lack of vision, congenitally-blind people need to rely more on their remaining senses, such as audition, touch or proprioception. It has been shown that extensive use of remaining sensory modalities can result in superior performance of congenitally-blind people, compared to sighted people, in perceptual, spatial, and attentional tasks [43], [44]. Therefore, congenitally blind participants may have effectively used the available auditory and tactile information provided by the co-actor, i.e., they used an agent-based reference frame, in order to build up a more reliable spatial target representation than they would by using response-based coding alone. In particular, in the hands-crossed condition where the arm of the co-actor lay over the arm of the actor, the direct tactile feedback of the co-actor's arm might have increased the saliency of that person's location and, thus, the use of an agent-based reference frame.

Alternatively, the results that were predicted for agent-based coding might also be explained by the use of an internal, hand-centered reference frame. The study by Röder et al. [9] on individual action control demonstrated that congenitally-blind people code the location of a stimulus relative to the anatomical origin of their responding hands, i.e., the Simon effect reversed when the left hand operated the right response key and vice versa (crossed hand condition). If congenitally-blind participants had applied such an internal, hand-centered reference frame in the social Simon task (i.e., referencing the stimulus location to the position of one's own hand, regardless of the location of the response key), one would have expected a similar result pattern as for agent-based coding, but only for the person sitting on the right operating the response key with his/her right hand. That is, for the person sitting on the right side, a positive SSE should occur under uncrossed hands (right hand on right key) and a negative SSE should occur under crossed hands (right hand on left key). Importantly, this pattern would be reversed for the left-sitting participant (i.e., negative SSE for uncrossed hands with right hand on left key and positive SSE for crossed hands with right hand on right key; cf. Fig. 1, right panel). We tested this hypothesis by conducting an additional ANOVA with Compatibility, Condition and Sitting Position (left, right) to the congenitally-blind group and found neither a significant main effect (*F*_1,14_<1) nor any significant interaction with Sitting Position (all *F*'s<1). This finding rules out the use of an internal, observer-centered reference frame and, therefore, favors the use of external reference frames by congenitally-blind people during social interaction.

Further, our findings challenge the action co-representation account [16], [35], which would have predicted comparable task performance for crossed and uncrossed hand positions (when S–R compatibility is constantly coded with respect to the location of the responding hand), given that participants represent their own and others' actions in a functional equivalent way [16]. While the present results of the sighted people are perfectly in line with these assumptions (i.e., an overall positive SSE irrespective of hand position), the results of the congenitally-blind participants are inconsistent with the action co-representation account (i.e., an overall positive SSE when hands are uncrossed and no SSE when hands are crossed). According to the referential coding account [23]-[24], however, one's own action is coded as left or right with respect to some point of reference (another person or object producing action events). If this left–right code is shared by a stimulus, the processing of this stimulus will prime the corresponding response [24]. However, action codes represent the features of all perceivable action effects [14], [57]–[58]. In an auditory social Simon task, blind people perceive spatially distributed auditory stimuli, auditory and tactile feedback of their own responses, and auditory feedback of the other person's response. In the hands-crossed condition the relative location of the other agent's body position is additionally represented by means of the tactile feedback of the crossed arms. Moreover, the auditory feedback of the other person's response is perceived in front of one's own body and the auditory feedback of one's own action is perceived in the location of the other agent's body when hands are crossed. This produces an enormous amount of conflicting information related to agent- and response-based coordinates in the social Simon task that is absent when performing the task alone (e.g., in the standard Simon task). While constant visual feedback (sighted participants) or enormous experience with response-based coding (blindfolded participants) may compensate for interfering signals in such high-complex situations, such a default strategy is non-applicable for congenitally-blind people. In order to reduce the conflict, they instead seem to use a more flexible strategy of response-based and agent-based coding, depending on the compatibility of the previous trial. Even though further research is needed to improve our understanding of the contextual dependencies and inter-individual abilities that may determine different strategies for coordinating actions in time and space, we take the present findings as support for the referential coding account [23]–[24], which can explain the data of both the sighted and the congenitally-blind individuals.

To summarize, the present study extends previous findings on individual action control [9]–[10] by demonstrating the default use of external coding strategies during joint action, independent of the presence or (transient/permanent) absence of vision. Hence, changing the contextual demands of action control from individual to joint task performance seems to strengthen the reliance on external reference frames. Moreover, our findings suggest that visual experience determines the type of external reference frame/s (response-based vs. agent-based) used in social manual tasks. While response-based coding seems to be the preferred coding strategy when visual input is available during ontogeny, response-based coding is combined with agent-based coding when vision is completely lacking since birth.
